# Text Messaging Interventions for Reducing Alcohol Consumption Among Harmful and Hazardous Drinkers: Protocol for a Systematic Review and Meta-Analysis

**DOI:** 10.2196/12898

**Published:** 2019-04-23

**Authors:** Marcus Bendtsen

**Affiliations:** 1 Department of Medical and Health Sciences, Linköping University Linköping Sweden

**Keywords:** text messaging, SMS, risky drinking, harmful drinking, hazardous drinking, intervention, systematic review, meta-analysis

## Abstract

**Background:**

Mobile phone-based interventions have become popular for lifestyle behavior change, particularly the use of text messaging as it is a technology ubiquitous in mobile phones. Reviews and meta-analyses of digital interventions for reducing harmful and hazardous use of alcohol have mainly focused on Web-based interventions; thus, there is a need for a body of evidence to guide health practitioners, policy makers, and researchers with respect to the efficacy of available text messaging interventions.

**Objective:**

The aim of this systematic review and meta-analysis is to assess the effectiveness of text messaging interventions for reducing the amount of alcohol consumed among harmful and hazardous drinkers; this is compared to receiving no, minimal, or unrelated health information. Specifically, we ask the following questions: (1) Can interventions consisting of only text messages be effective in reducing alcohol consumption compared to no intervention or a minimal or unrelated intervention? (2) Can interventions consisting of only text messages be effective in reducing the prevalence of risky drinking compared to no intervention or a minimal or unrelated intervention?

**Methods:**

Several databases will be searched, including the Cochrane Central Register of Controlled Trials (CENTRAL), MEDLINE, PsycINFO, the Conference Proceedings Citation Index, ClinicalTrials.gov, OpenGrey, among others. Reports of studies that evaluate text messaging interventions for reducing the amount of alcohol consumed will be included. Primary outcomes of interest will be weekly alcohol consumption and frequency of heavy episodic drinking. The Cochrane Collaboration Risk of Bias tool will be used to assess bias in reports, and the Grades of Recommendation, Assessment, Development, and Evaluation (GRADE) approach will be used to assess the quality of the body of evidence. A narrative review will be presented, and a meta-analysis will be conducted in case of homogeneity among included studies.

**Results:**

The systematic review has not yet begun but is expected to start in May of 2019; publication of the final review and meta-analysis is expected at the end of 2019.

**Conclusions:**

The technology for text messaging is ubiquitous in mobile phones; thus, the potential reach of interventions utilizing this technique is great. However, there are no meta-analyses to date that limit the scope to the use of text messaging interventions for alcohol consumption reduction. Therefore, the proposed systematic review and meta-analysis will help health practitioners, policy decision makers, researchers, and others to better understand the effects of these interventions.

**International Registered Report Identifier (IRRID):**

PRR1-10.2196/12898

## Introduction

### Rationale

Hazardous drinking, defined as a quantity or pattern of consumption that places an individual at risk for adverse health events [1], is a modifiable behavior that increases the risk of noncommunicable diseases [2]. Noncommunicable diseases are responsible for 70% of deaths globally each year, of which cardiovascular diseases, cancer, respiratory diseases, and diabetes account for over 80% [3,4]. Recent evidence suggests that alcohol consumption at any level increases the risk of stroke, heart failure, fatal hypertensive disease, fatal aortic aneurysm, and coronary disease, excluding myocardial infarction, but may on the other hand have a protective effect on myocardial infarction [5].

Harmful drinking, defined as consumption of alcohol that results in physical or psychological harm [1], may lead to injury, road traffic accidents, violence, and social and economic burdens, as well as having a causal relationship with a range of mental and behavioral disorders [6]. Harmful use of alcohol contributes to 5.9% of deaths globally, and as much as 25% of total deaths in the age group 20-39 years are attributable to harmful drinking [2,7]. Furthermore, harmful use of alcohol is the seventh-leading risk factor for disability-adjusted life years and is the leading risk factor of death among those aged 15-49 years [8].

### mHealth and Text Messaging

The World Health Organization (WHO) defines eHealth as the use of information and communication technologies for health, including electronic health records, patient management systems, ecological monitoring, robotics, lab systems, informatics, etc. By extension, eHealth interventions can be understood as interventions that promote health using information and communication technologies. With the global growth of mobile phone subscriptions—in 2016, it was estimated that 95% of the global population resided in an area with a mobile-cellular network [9]—a subfield of eHealth called mHealth (ie, mobile health) has emerged [10]. Continuous contact with individuals, interactivity, and cost reductions are only some of the benefits that mHealth interventions may be associated with.

Text messaging, more formally known as short message service (SMS), is a technology ubiquitous in mobile phones. The technology does not rely on data networks such as 3G or 4G, which usually incur extra costs for end users and may be unavailable in certain areas, but runs on networks utilizing earlier standards such as Global System for Mobile communications (GSM), which are generally more available and cheaper. Thus, there is potentially a great reach for lifestyle interventions that utilize text messaging.

Trials of text messaging for smoking cessation have shown positive results [11-13], and a Cochrane review reported a beneficial impact of mobile phone-based smoking cessation programs [14], most of which were text message based. However, while there have been evaluations of text messaging interventions for alcohol consumption reduction [15-19], there have been no meta-analyses regarding the use of text messaging programs for reducing alcohol consumption, despite the need to collect evidence and provide guidance on these types of interventions.

### Digital Interventions for Alcohol Reduction

Previous meta-analyses of digital interventions targeting harmful and hazardous use of alcohol have mainly focused on trials of electronic screening and brief interventions [20-23]. Commonly, individuals engaging with this type of intervention respond to a series of questions, after which a summary of their alcohol habits is presented; feedback is then given with respect to recommended drinking levels, alongside some advice on behavior change. The focus was broadened in a Cochrane review to include all digital interventions [24], which identified moderate-quality evidence that digital interventions may lower alcohol consumption; however, the authors emphasize that “Most included trials tested Web‐based interventions, so the effectiveness of other types of interventions such as smartphone apps or SMS messages is less clear.” Thus, there still exists a knowledge gap with respect to the efficacy of text messaging interventions. A recent systematic review [25] supported the use of mHealth interventions to address substance use; however, while included trials of interventions were delivered in a variety of formats (eg, Web based, text messaging, and mobile phone apps), overall effectiveness in a meta-analysis was not quantified.

### Objectives

The aim of this systematic review and meta-analysis is to assess the effectiveness of text messaging interventions for reducing the amount of alcohol consumed among harmful and hazardous drinkers; this is compared to receiving no, minimal, or unrelated health information. Specifically, we ask the following questions:

1. Can interventions consisting of only text messages be effective in reducing alcohol consumption compared to no intervention or a minimal or unrelated intervention?
2. Can interventions consisting of only text messages be effective in reducing the prevalence of risky drinking compared to no intervention or a minimal or unrelated intervention?

## Methods

### Protocol Registration and Development

In accordance with the guidelines, this systematic review protocol was registered with the International Prospective Register of Systematic Reviews (PROSPERO) on April 3, 2019 (registration number: CRD42019117431). The Preferred Reporting Items for Systematic Reviews and Meta-Analyses Protocols (PRISMA-P) statement [26] has been followed when developing this protocol. The execution and reporting of the described systematic review and meta-analysis will be done in line with the Preferred Reporting Items for Systematic Reviews and Meta-Analyses (PRISMA) statement [27].

### Eligibility Criteria

#### Study Design

We will include randomized controlled trials (RCTs), including cluster RCTs.

#### Participants

Studies examining harmful or hazardous drinkers identified by a screening tool in any population (eg, students, general population, and primary care patients) will be included. No restriction on age or population will be made, as the review attempts to assess the effect of text messaging among any harmful or hazardous drinkers. However, studies that include participants who are obviously receiving care for their alcohol consumption (eg, patients in a treatment program) will not be included. The aim is to assess the effect of text messaging interventions as the main intervention, not as a cointervention to other treatment programs. Recruitment can be done through different means (eg, through email, workplaces, at emergency rooms, and in primary care settings). However, studies will be excluded if participants were mandated to take part in the trial.

#### Interventions

Interventions of interest should consist of a series of text messages sent to participants’ mobile phones over a number of weeks. For an intervention to be included, at least two messages should be sent per week, on average. The content of the messages should be focused on behavior change, thus excluding studies where text messages are used only to schedule or remind participants of other activities. Only studies where a text message intervention is the sole intervention will be considered; therefore, studies of interventions where text messages are combined with other interventions (eg, therapy or pharmaceutical treatment) will be excluded.

In cases where the intervention targets multiple behaviors or conditions (eg, smoking and depression), the study report will be included if participants were screened into the trial (ie, nonharmful and nonhazardous drinkers were excluded) and alcohol consumption outcomes are available.

#### Comparators

Four types of control settings will be considered: 

1. Minimal contact and potentially put on a waiting list. For instance, this includes participants allocated to the control setting that were told they would not be given access to the novel intervention, and possibly that they would be given access once the trial was finished.
2. Basic health information. For instance, this includes participants allocated to the control setting that were given basic information about the risks of alcohol at the time of randomization and possibly contacted with similar information at intervals throughout the period, however, at a lower frequency than twice a week, on average.
3. Referred to self-help. For instance, this includes participants allocated to the control setting that were told that they should access a website for more information and help or that they should contact their primary health care provider, etc. Additional contact in the form of reminders at a lower frequency than twice a week, on average, is acceptable.
4. Intervention focusing on something other than alcohol consumption. For instance, this includes participants in the control setting that were given an intervention with content about physical activity, smoking, nutritional intake, etc.

#### Outcomes

Studies will be included if they measured one of two common alcohol consumption outcomes:

1. Weekly alcohol consumption measured in grams of alcohol. If the outcome is reported in terms of standard units, then it will be converted to grams of alcohol based on the definition reported in the study, or inferred based on the country in which the trial was run.
2. Number of episodes of heavy drinking during the past month. Cutoff points for heavy episodic drinking may differ; however, cutoff points commonly sit at 3 (female) or 4 (male) standard units of alcohol on a single occasion. We will adopt the cutoffs used in the respective studies.

#### Timing

Length of follow-up will be defined based on time elapsed since randomization. Studies will not be excluded based on the timing of follow-up.

#### Language

We will include reports in English.

### Information Sources

We will search for literature in PubMed, including MEDLINE and PubMed Central; Cochrane Central Register of Controlled Trials (CENTRAL); Cochrane Database of Systematic Reviews (CDSR); Database of Abstracts of Reviews of Effects (DARE); National Health Service Economic Evaluation Database (NHS-EED); Scopus; PsycINFO; PsycARTICLES; Cumulative Index to Nursing and Allied Health Literature (CINAHL); Science Citation Index (ie, Web of Science); Social Sciences Citation Index (ie, Web of Science); and Conference Proceedings Citation Index (ie, Web of Science). Journals in which included literature from the electronic databases are published will be searched, and reference lists of the included studies will be scanned for additional literature.

The following clinical trial registries will be searched: International Standard Randomised Controlled Trial Number (ISRCTN) registry; ClinicalTrials.gov; and the WHO International Clinical Trials Registry Platform (ICTRP). Grey literature will be sourced from the OpenGrey database. PROSPERO will also be searched to identify completed, ongoing, or planned systematic reviews and meta-analyses of relevance. Reports included in any relevant systematic reviews and meta-analyses will also be searched. Finally, authors’ personal files will be consulted.

### Review Team

MB is the guarantor. A review team consisting of at least three researchers, including MB, will be put together before the search stage begins. The review team will consist of individuals with extensive experience in the development and evaluation of lifestyle interventions.

**Draft of search strategies for PubMed.**

Search strategies for PubMed articles:

Strategy 1: (randomized controlled trial [pt] OR controlled clinical trial [pt] OR randomized [tiab] OR placebo [tiab] OR drug therapy [sh] OR randomly [tiab] OR trial [tiab] OR groups [tiab])

Strategy 2: (animals [mh] NOT humans [mh])

Strategy 3: (Alcohol* OR Risky Drink* OR Harmful Drink* OR Hazardous Drink* OR Heavy Episodic Drink*)

Strategy 4: mobile* OR mobile phone* OR cell* OR cell phone*

Strategy 5: Text Messaging [mh] OR Text Messag* OR Mobile Messag* OR SMS OR Short Messag* OR Texts

Strategy 6: Strategy 1 NOT Strategy 2

Strategy 7: Strategy 3 OR Strategy 4 OR Strategy 5

Strategy 8: Strategy 6 AND Strategy 7

### Search Strategy

A strategy for PubMed will be developed. Once this strategy is finalized, it will be translated to the syntax for the other databases. Medical Subject Headings (MeSH) terms and text words related to alcohol, harmful and hazardous drinking, text messaging, etc, will be used in the search strategies. The Cochrane Highly Sensitive Search Strategy for identifying randomized trials in MEDLINE: sensitivity-maximizing version (2008 revision) [28], will be used to filter RCTs. A draft of the search strategies can be found in Textbox 1.

### Study Records

#### Data Management and Selection Process

Search results will be input into Mendeley, the reference management software. Initially, the guarantor (MB) will screen the titles and abstracts for duplicates and will also remove reports of studies that are clearly deemed irrelevant for the objective. Reports of studies for which uncertainty exists regarding their relevance will be kept at this stage; removed reports will be shown to the rest of the research team to confirm that nothing has been removed that should be considered for inclusion. Each member of the research team will then independently analyze the full text of the retained studies, deciding which studies to include using the eligibility criteria. If necessary, report authors will be contacted for further information. Disagreements that cannot be resolved will be arbitrated by the guarantor. Excluded and removed studies will be stored for future reference along with an explanation for why they were not included.

#### Data Collection Process

A data form in MS Excel will be used to record extracted data for each included trial. Each member of the research team will extract data independently. Differences will be discussed among team members and arbitrated by the guarantor, possibly after contacting report authors for further details.

### Data Items

#### Overview

The following quantitative items will be extracted from the trials:

1. Alcohol consumption measures at baseline and follow-up: mean or median and dispersion for weekly alcohol consumption and heavy episodic drinking. Prevalence of risky drinking according to national guidelines.
2. Characteristics of the randomized individuals: for example, age and gender.
3. Trial procedures: for example, number randomized, group sizes, number of responses, trial design, and duration of follow-up.
4. Details of intervention: for example, number of weeks the intervention lasted and average weekly frequency of text messages.

The following qualitative items will be extracted from the trials:

1. Control: the type of control setting used in the trial.
2. Support: the type and source of financial support.

If necessary, outcomes will be extracted or approximated from figures in the reports. We will, as far as possible, extract data from intention-to-treat analyses and remark on trials that do not report on these.

#### Data Simplifications

Some trials may consist of more than two arms (eg, comparing variations of a text messaging intervention with a single control group). If only one arm fit the eligibility criteria for the proposed review, then we will extract only this arm and the control group. If multiple arms fit the criteria, then they will be combined into one arm, so as to avoid multiple comparisons with the same control group. Likewise, if multiple control groups are utilized with very similar control settings, then they will be combined (ie, weighted mean for continuous outcomes and summing dichotomous outcomes).

### Outcomes and Prioritizations

#### Primary Outcomes

There are two primary outcomes: weekly alcohol consumption and heavy episodic drinking.

##### Weekly Alcohol Consumption

Reports of weekly alcohol consumption will likely be self-reported via questionnaires or interviews. We expect that two modes of assessing alcohol consumption will be in the majority: either a look-back period approach or a frequency-intensity approach. Regardless of the method used, we will convert the units to grams per week for each study, an approach used in previous meta-analyses where weekly alcohol consumption has been an outcome.

##### Heavy Episodic Drinking

Heavy episodic drinking will also likely be self-reported, however, there is usually more homogeneity in how this is collected. Typically, individuals are asked to report the number of times they drank more than 3 (female) or 4 (male) standard units of alcohol on the same occasion over the past month. Some countries include a time period during which the units should have been consumed, however, we will not take any action to account for this difference. What also may differ is the use of fixed-response options (eg, *Once or twice a week*) or a numerical measure. We will convert all data to monthly assessments, converting fixed-response options to numerical measures by taking mean values and multiplying appropriately, for example, *Once or twice a week* would be (1 + 2)/2 × 4 = 6.

#### Secondary Outcome

The single secondary outcome is prevalence of risky drinking. Definitions vary between countries; however, risky drinking is typically defined as drinking in excess of 7 (female) or 14 (male) standard units of alcohol per week or one or more heavy episodes of drinking per month. The definition of a standard unit differs between countries and typically ranges from 8 to 14 grams of ethanol per unit. We will adopt the definition used in each respective trial.

#### Risk of Bias in Individual Studies

To assess the risk of bias in each trial, two team members will independently collect information using the Cochrane Collaboration’s tool for assessing risk of bias [28,29]. Differences will first be discussed; if no consensus is found, then a third team member will arbitrate. The tool assesses several sources of bias, including selection, performance, detection, attrition, and reporting. This is done by judging the sequence generation, allocation sequence concealment, blinding of participants and personnel, blinding of outcome assessment, incomplete outcome data, selective outcome reporting, etc. The assessment will result in a *low risk*, *high risk*, or *uncertain risk* classification of each included trial.

The publication in which each included trial was initially published will be scrutinized for predatory behavior, since predatory publications usually apply less rigor in their review practices. If a publication is found to be predatory, either through Cochrane’s list of predatory publications or the website Stop Predatory Journals [30], then we will consider removing the trial completely or classifying the trial as *high risk*.

### Data Synthesis

#### Overview

A systematic narrative synthesis will be presented in line with the guidance from the Centre for Reviews and Dissemination [31]. A meta-analysis will be conducted using a random-effects model if at least two included trials are found to be sufficiently homogenous with respect to trial design, intervention, and comparator.

1. The two primary continuous outcomes—weekly consumption and heavy episodic drinking—will be analyzed using weighted mean differences with 95% CI. Transformed data will be back-transformed, and reports of medians will be taken as the best approximator of the mean; ranges will be converted to standard deviations, as described in the Cochrane Handbook for Systematic Reviews [28].
2. The secondary dichotomous outcome will be analyzed in terms of relative risk with 95% CI and pooled in a meta-analysis using Mantel-Haenzel weighting.

Since individuals randomized is the primary unit of analysis, the interclass correlation coefficient will be extracted from reports on cluster randomized trials and results modified according to the Cochrane Handbook for Systematic Reviews.

In studies where outcomes have been assessed more than once, we will use data from the first postintervention analysis. For instance, an intervention might last for 12 weeks and have assessments at 6, 12, and 18 weeks. In this case, the 12-week assessment will be the primary assessment in the meta-analysis. However, all data will be extracted, since subgroup analyses will be conducted for different time frames.

#### Subgroup and Sensitivity Analyses

If outcome data are available, subgroup analyses will be conducted with respect to age, creating three equal-width categories, and gender. Also, separate analyses will be created for pragmatic groupings of follow-up periods; for instance, we may find that we can define three periods, such as 0-3 months (short), 4-6 months (mid), and 7+ months (long); however, we will leave the exact time frames undefined at this stage.

Sensitivity analyses will be conducted by exploring the effect of removing trials that do not report intention-to-treat analyses, are at high risk of bias due to follow-up attrition or otherwise missing data, have been classified as having a high or uncertain risk of bias due to allocation concealment, have had standard deviation imputed, or employ cluster randomization.

#### Assessment of Heterogeneity and Publication Bias

Heterogeneity magnitude among trials will be assessed using the I^2^ statistic, and significance will be assessed using χ^2^ tests (significance level .1). If heterogeneity is found using the Cochrane recommended cutoffs for the I^2^ statistic [28], we will explore sources for the heterogeneity. Publication bias assessment using funnel plots will only be considered if at least 10 studies are included. Trial registration databases and protocols will be consulted to ensure that recruitment began after registration and publication of protocols and that the trial conformed to the study design and analysis planned.

#### Confidence in Cumulative Estimate

By only including RCTs, we will aim for the highest-quality rating according to the Grades of Recommendation, Assessment, Development, and Evaluation (GRADE) approach [28,32]. The highest grade should be given to bodies of evidence that instill confidence that an estimate of effect is close to the quantity of interest. However, downgrading this rating will be necessary after evaluating quality ratings of each outcome, which includes assessment of factors such as imprecision of results and unexplained heterogeneity. We will report on a final grade of the evidence collected.

## Results

The systematic review has not yet begun but is expected to start in May of 2019; publication of the final review and meta-analysis is expected at the end of 2019.

## Discussion

The technology for text messaging is ubiquitous in mobile phones; thus, the potential reach of interventions utilizing this technique is great. However, there are no systematic reviews to date, as far as the authors are aware, that limit the scope to the use of text messaging interventions for alcohol consumption reduction. Therefore, the proposed systematic review and meta-analysis will help health practitioners, policy decision makers, researchers, among others, to better understand the effects of these interventions.

Due to the narrowness of the research question, it is likely that only a few studies will be available for inclusion in the proposed review. However, the final review should be interpreted as a summary of current evidence and will be updated when new evidence is made available. The narrative review and meta-analysis is planned to be updated every 2 years.

## Abbreviations

CDSR: Cochrane Database of Systematic Reviews
CENTRAL: Cochrane Central Register of Controlled Trials
CINAHL: Cumulative Index to Nursing and Allied Health Literature
DARE: Database of Abstracts of Reviews of Effects
GRADE: Grades of Recommendation, Assessment, Development, and Evaluation
GSM: Global System for Mobile communications
ICTRP: International Clinical Trials Registry Platform
ISRCTN: International Standard Randomised Controlled Trial Number
MeSH: Medical Subject Headings
NHS-EED: National Health Service Economic Evaluation Database
PRISMA: Preferred Reporting Items for Systematic Reviews and Meta-Analyses
PRISMA-P: Preferred Reporting Items for Systematic Review and Meta-Analysis Protocols
PROSPERO: Prospective Register of Systematic Reviews
RCT: randomized controlled trial
SMS: short message service
WHO: World Health Organization
